# Supplementary material for: Chiral self-sorted multifunctional supramolecular biocoordination polymers and their applications in sensors
Source: Nat Commun. 2018 Sep 26;9:3933. doi: 10.1038/s41467-018-06147-8 (PMC6158292; doi:10.1038/s41467-018-06147-8)
Supplement: Supplementary file 1 — Supplementary Information [file 41467_2018_6147_MOESM1_ESM.pdf]

Supplementary Information:

Chiral self-sorted multifunctional supramolecular biocoordination polymers and their applications in sensors

Shang et al.

Supplementary Methods

Measurement of optoelectrical properties

In order to investigate photosensitivity of photodetectors, photoresponsivity (R) and photocurrent/dark-current ratio (P) were calculated from *I-V* characteristics coupled with light irradiation. The R and P values are typically defined by the following equations:

$$R = \frac{I_{ph}}{P_{inc}} = \frac{I_{light} - I_{dark}}{P_{inc}}, \quad (S1)$$

$$P = \frac{I_{light} - I_{dark}}{I_{dark}}, \quad (S2)$$

where I_{ph} is the photocurrent, P_{inc} the incident illumination power on the channel of the device, I_{light} the current under illumination, and I_{dark} the current in the dark, respectively. In addition, the external quantum efficiency (EQE, η) can be defined as the ratio of the number of photogenerated carriers that practically enhances the current to the number of photons incident onto the device channel area, using the following equation:

$$\eta = \frac{(I_{light} - I_{dark})hc}{eP_{inc}A\lambda_{peak}}, \quad (S3)$$

where h is the plank constant, c the speed of light, e the fundamental unit of charge, A the area of the device channel, and λ_{peak} the peak wavelength of the incident light, respectively.

Furthermore, detectivity (D^*) is an important figure of merit for photodetectors which usually describes the smallest detectable signal, allowing for the comparison between photodetector devices with different configurations and area. D^* was estimated for photodetectors using following equations:

$$D^* = \frac{\sqrt{A}}{NEP}, \quad (S4)$$

$$NEP = \frac{\sqrt{\overline{I_n^2}}}{R}, \quad (S5)$$

where A is the active area of the photodetectors, NEP the noise equivalent power, and $\overline{I_n^2}$ the measured noise current. If the shot noise from the current under dark condition is the major contribution to the noise limiting the detectivity, the D^* can be simplified as:

$$D^* = \frac{R}{\sqrt{(2e \cdot I_{dark}/A)}}, \quad (S6)$$

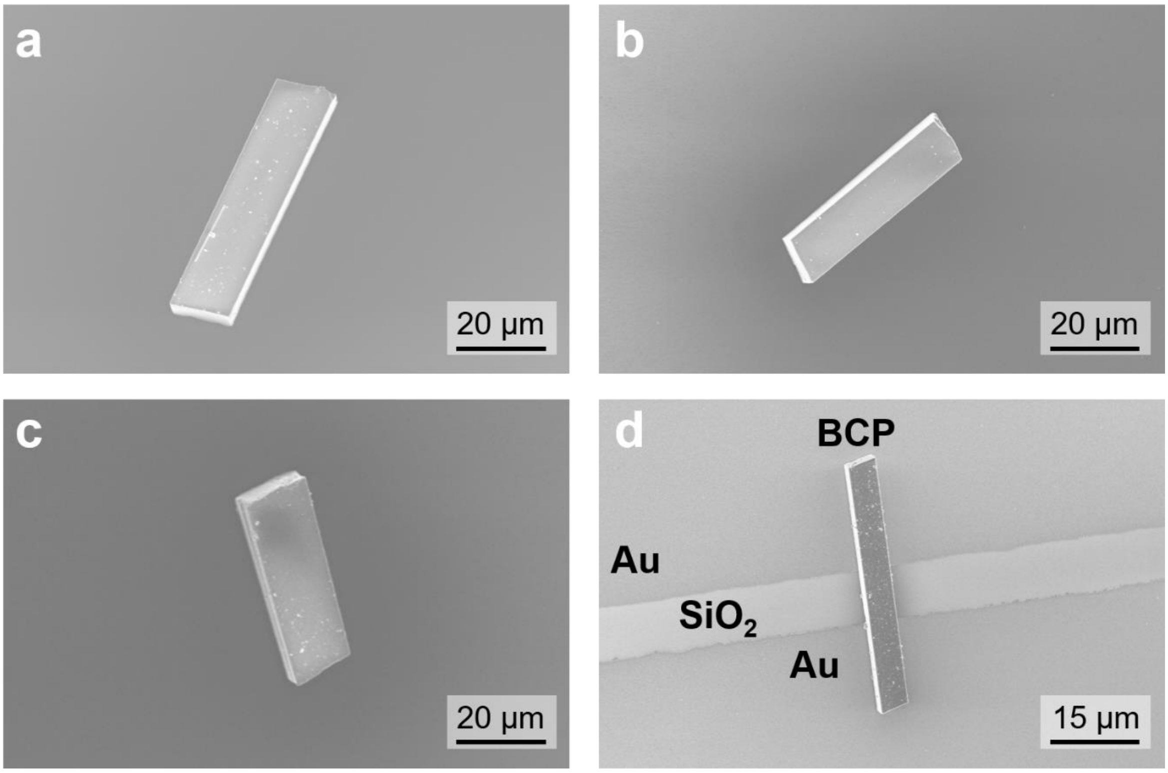

Supplementary Figure 1 | SEM images of AlaNDI-Zn SBCPs. SEM images of (**a**) (*R*)-AlaNDI-Zn, (**b**) (*S*)-AlaNDI-Zn, (**c**) (*Rac*)-AlaNDI-Zn SBCPs, and (**d**) (*Rac*)-AlaNDI-Zn SBCPs on pre-patterned gold electrodes in electronic devices.

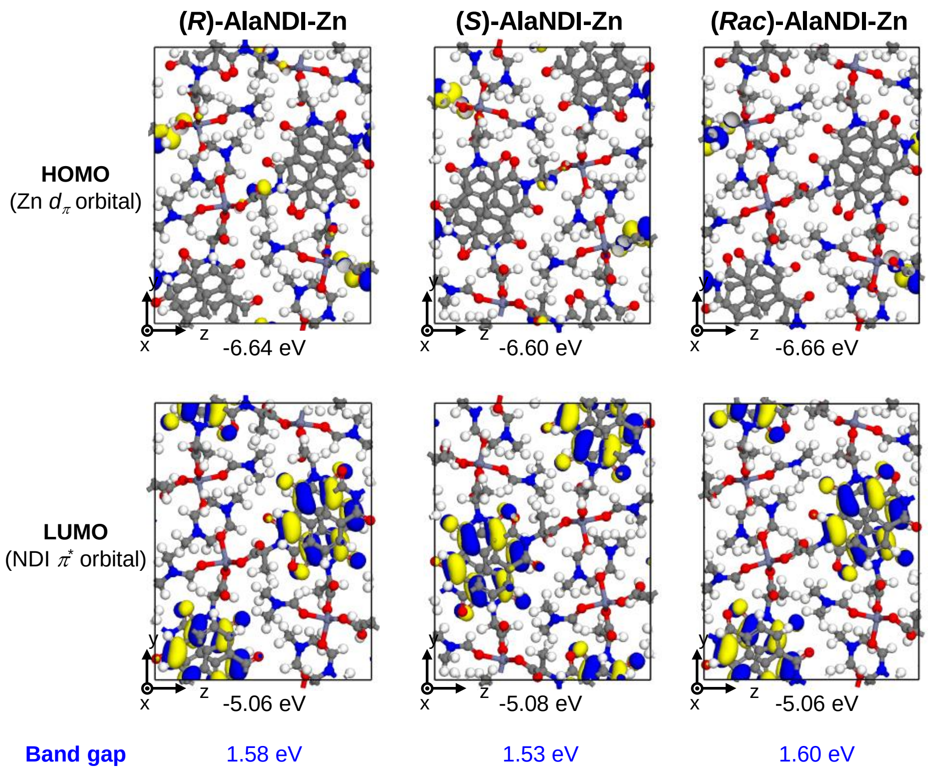

Supplementary Figure 2 | Energy level comparison of unit cells for (*R*)-, (*S*)-, and (*Rac*)-AlaNDI-Zn SBCPs. The carbon, hydrogen, oxygen, nitrogen and zinc atoms of (*Rac*)-AlaNDI-Zn are colored in gray, white, red, blue and thin-purple, respectively.

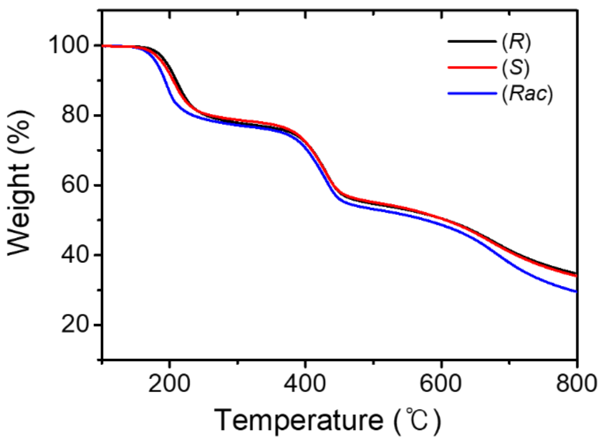

Supplementary Figure 3 | TGA results of homochiral and heterochiral AlaNDI-Zn SBCPs. TGA measurement results of homochiral and heterochiral AlaNDI-Zn SBCPs in nitrogen atmosphere.

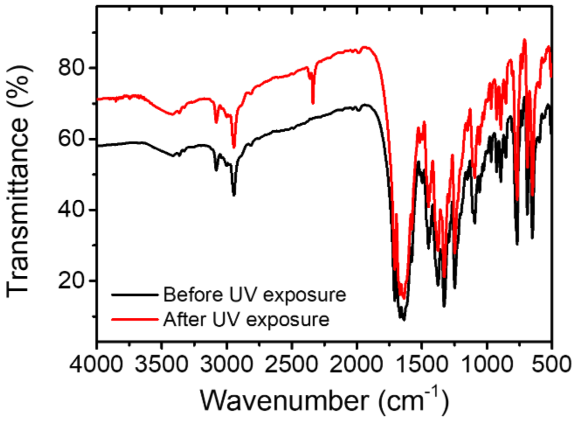

Supplementary Figure 4 | FT-IR spectra of heterochiral AlaNDI-Zn SBCPs before and after exposure to UV light. FT-IR spectra of (*Rac*)-AlaNDI-Zn before and after exposure to UV-light for 1 h (λ = 365 nm) (150 µW cm^{-2}).

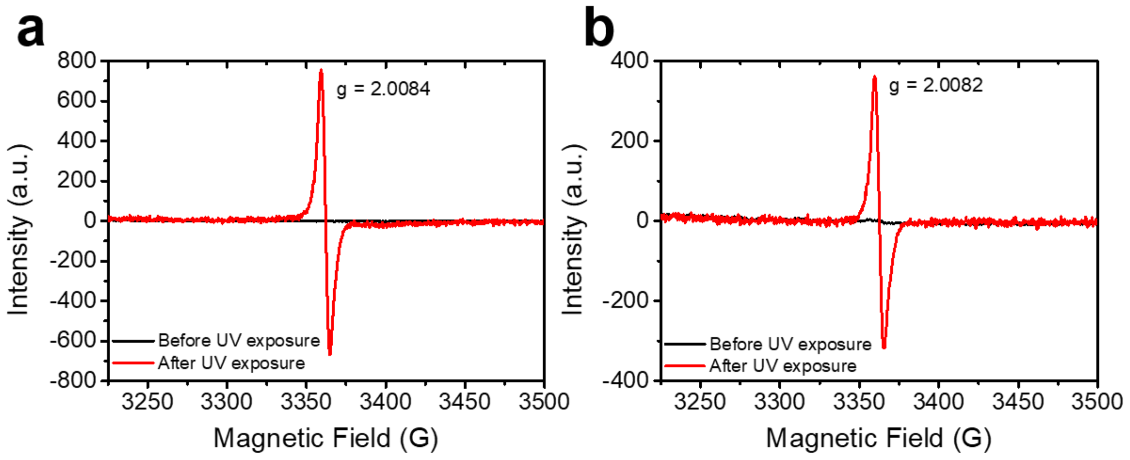

Supplementary Figure 5 | ESR spectra of homochiral AlaNDI-Zn SBCPs before and after exposure to UV light. (**a**,**b**) ESR spectra of (**a**) (*S*)-AlaNDI-Zn and (**b**) (*R*)-AlaNDI-Zn before and after exposure to UV light (λ = 365 nm) (150 μW cm^{-2}) for 1 h.

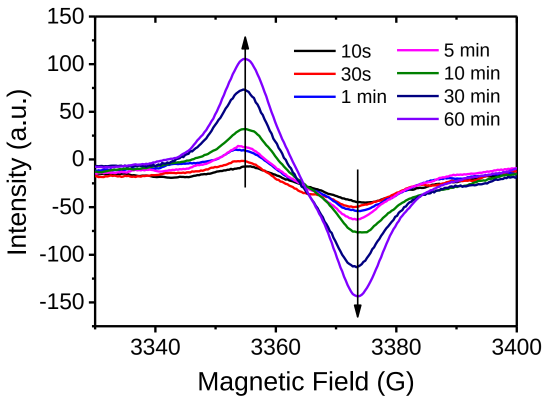

Supplementary Figure 6 | ESR spectra of heterochiral AlaNDI-Zn SBCPs depending on the UV light exposure time. ESR results of (*Rac*)-AlaNDI-Zn upon UV light irradiation depending on the exposure time.

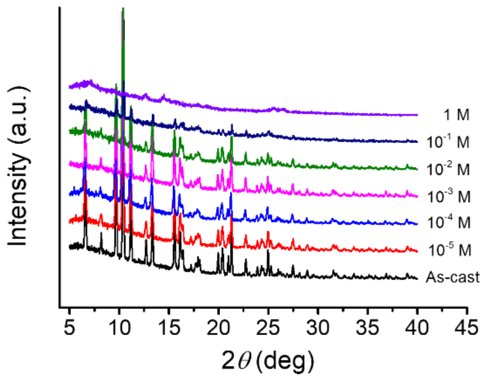

Supplementary Figure 7 | PXRD results of heterochiral SBCPs upon exposure to different concentrations of hydrazine. PXRD results of (*Rac*)-AlaNDI-Zn upon exposure to different concentrations of hydrazine solution in ethanol.

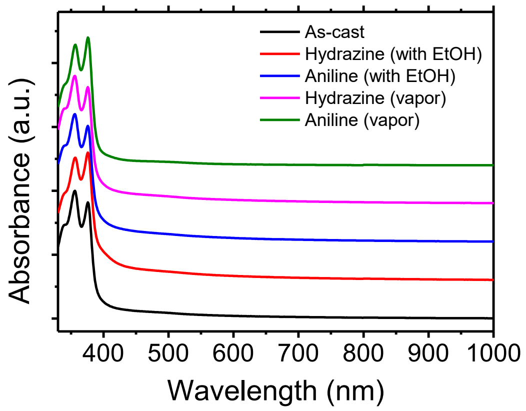

Supplementary Figure 8 | UV-Vis-NIR spectra of heterochiral AlaNDI-Zn SBCPs under exposure to hydrazine and aniline. UV-Vis-NIR spectra of (*Rac*)-AlaNDI-Zn in presence of hydrazine and aniline (0.1 M) in ethanol medium and after exposure to saturated hydrazine and aniline gas.

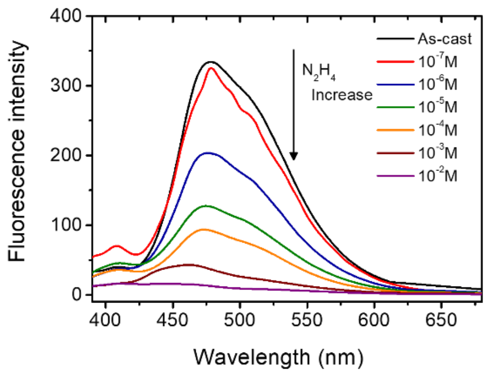

Supplementary Figure 9 | Quenching fluorescent intensity of homochiral (*S*)-AlaNDI-Zn SBCPs at different concentrations of hydrazine. Fluorescence intensity of (*S*)-AlaNDI-Zn in presence of different concentrations of hydrazine in EtOH at λ_{max} = 475 nm. (The excitation wavelength of 360 nm was chosen.)

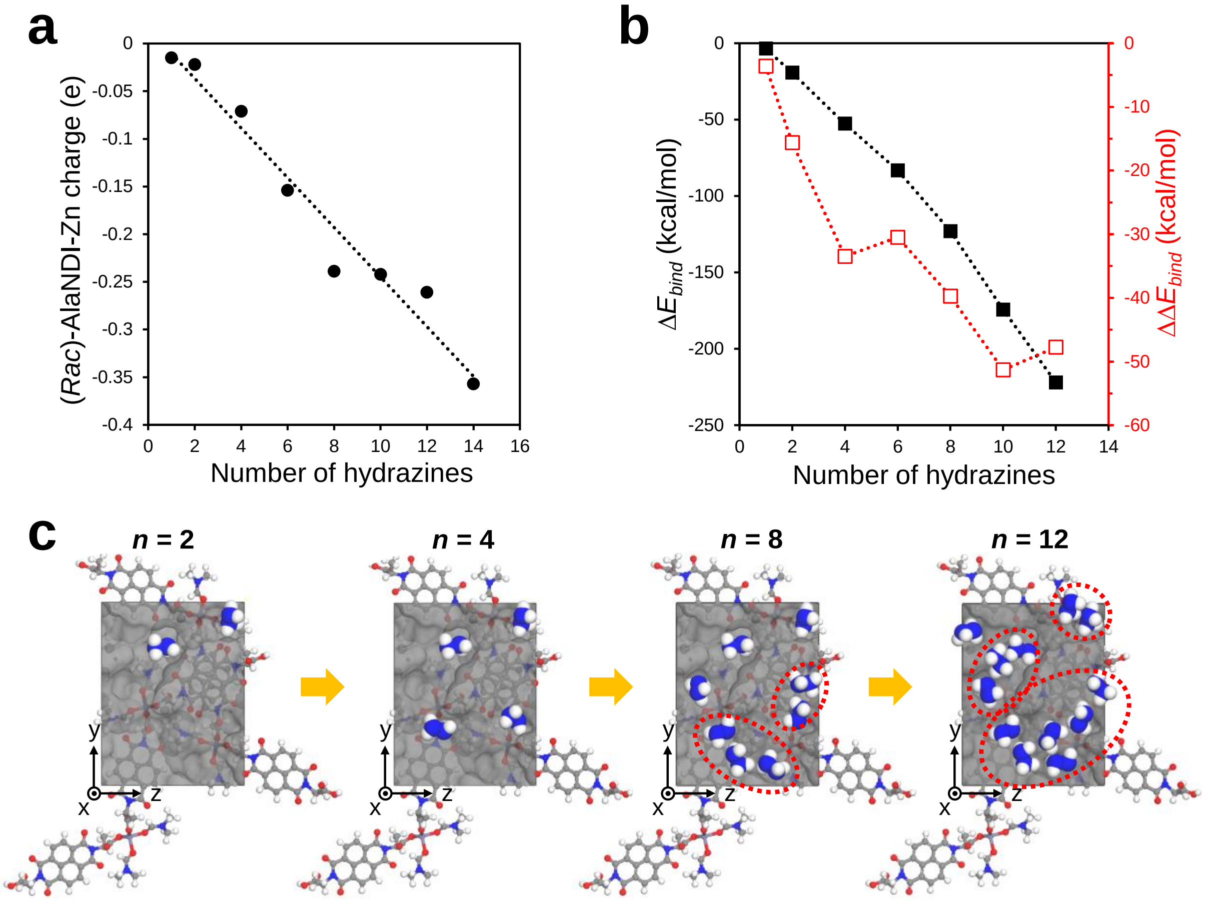

Supplementary Figure 10 | Charge transfer and binding interactions of hydrazine on heterochiral AlaNDI-Zn SBCP surface. (**a**) The accumulated negative charge of (*Rac*)-AlaNDI-Zn surface with increasing number of adsorbed hydrazine molecules obtained by Mulliken population analysis. (**b**) The binding energy (ΔE_{bind}) and differential binding energy ($\Delta\Delta E_{\mathrm{bind}}$) with increasing number of adsorbed hydrazine molecules. (**c**) Binding configurations of hydrazine molecules adsorbed on (*Rac*)-AlaNDI-Zn surface. The gray colored region represents the Connolly surface of (*Rac*)-AlaNDI-Zn for the clear view of the adsorbed configurations of hydrazine. The red dotted ovals represent the hydrogen bond formation between adjacent hydrazine molecules.

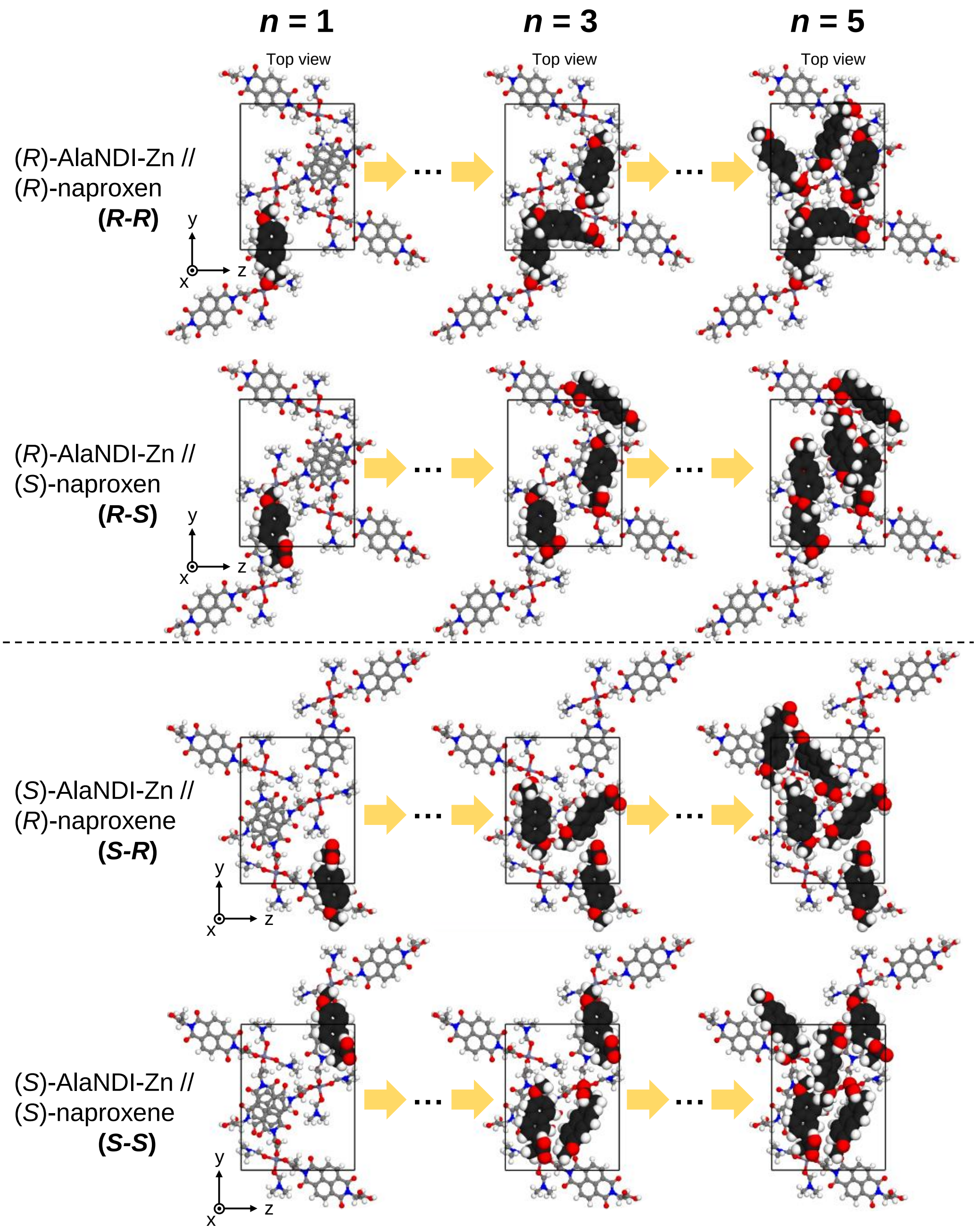

Supplementary Figure 11 | Binding configurations of chiral naproxen on homochiral AlaNDI-Zn SBCP surfaces. From top to bottom panel, the configurations of (*R*)- or (*S*)-naproxen adsorption on (*R*)-AlaNDI-Zn surface (*e.g.*, *R*-*R* or *R*-*S*), and (*R*)- or (*S*)-naproxen adsorption on (*S*)-AlaNDI-Zn surface (*e.g.*, *S*-*R* or *S*-*S*) are presented. The carbon, hydrogen, oxygen, nitrogen and zinc atoms of AlaNDI-Zn are colored in gray, white, red, blue and thin-purple, respectively. The naproxen molecules adsorbed on the surface are represented in CPK style (*i.e.*, C : black, O : red, H : white).

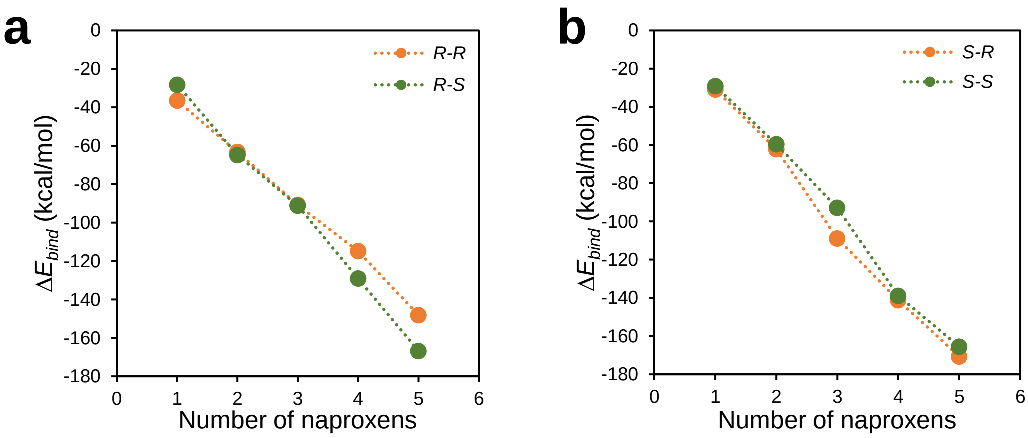

Supplementary Figure 12 | Binding energy (ΔE_{bind}) comparison of chiral naproxen on homochiral AlaNDI-Zn surfaces. (**a**) (*R*)- or (*S*)-naproxen adsorption on (*R*)-AlaNDI-Zn surface. (*e.g.*, *R*-*R* or *R*-*S*) (**b**) (*R*)- or (*S*)-naproxen adsorption on (*S*)-AlaNDI-Zn surface (*e.g.*, *S*-*R* or *S*-*S*).

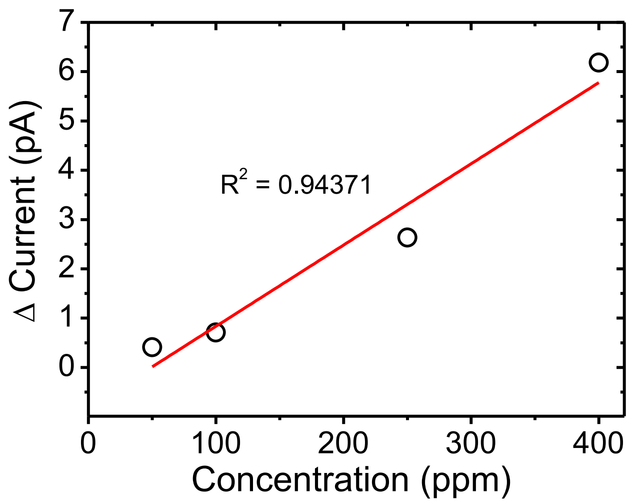

Supplementary Figure 13 | Linear plot of current change depending on aniline concentration. Linear plot of current change vs the concentration of aniline gas.
